# CRISPR loci-PCR as Tool for Tracking *Azospirillum* sp. Strain B510

**DOI:** 10.3390/microorganisms9071351

**Published:** 2021-06-22

**Authors:** Joaquin I. Rilling, Fumito Maruyama, Michael J. Sadowsky, Jacquelinne J. Acuña, Milko A. Jorquera

**Affiliations:** 1 Applied Microbial Ecology Laboratory (EMAlab), Departamento de Ciencias Químicas y Recursos Naturales, Universidad de La Frontera, Temuco 4780000, Chile; joaquin.rilling@ufrontera.cl; 2 Center of Plant, Soil Interaction and Natural Resources Biotechnology, Scientific and Technological Bioresource Nucleus (BIOREN), Universidad de La Frontera, Temuco 4780000, Chile; 3 Office of Industry-Academia-Government and Community Collaboration, Hiroshima University, Hiroshima 739-8511, Japan; fumito@hiroshima-u.ac.jp; 4 Department of Soil, Water, and Climate, Department of Plant and Microbial Biology, and BioTechnology Institute, University of Minnesota, St. Paul, MN 55812, USA; sadowsky@umn.edu

**Keywords:** *Azospirillum*, microbial inoculants, strain tracking methods, CRISPR_loci_-PCR

## Abstract

*Azospirillum*-based plant and soil inoculants are widely used in agriculture. The inoculated *Azospirillum* strains are commonly tracked by both culture-dependent and culture-independent methods, which are time-consuming or expensive. In this context, clustered regularly interspaced short palindromic repeats (CRISPR) loci structure is unique in the bacterial genome, including some *Azospirillum* species. Here, we investigated the use of CRISPR loci to track specific *Azospirillum* strains in soils systems by PCR. Primer sets for *Azospirillum* sp. strain B510 were designed and evaluated by colony and endpoint PCR. The CRISPR_loci_-PCR approach was standardized for *Azospirillum* sp. strain B510, and its specificity was observed by testing against 9 different *Azospirillum* strains, and 38 strains of diverse bacterial genera isolated from wheat plants. The CRISPR_loci_-PCR approach was validated in assays with substrate and wheat seedlings. *Azospirillum* sp. strain B510 was detected after of two weeks of inoculation in both sterile and nonsterile substrates as well as rhizosphere grown in sterile substrate. The CRISPR_loci_-PCR approach was found to be a useful molecular tool for specific tracking of *Azospirillum* at the strain level. This technique can be easily adapted to other microbial inoculants carrying CRISPR loci and can be used to complement other microbiological techniques.

## 1. Introduction

Biofertilizer products based on microbial inoculants have been marketed since the late 1800’s and are currently commercialized in many countries. It has been estimated that by 2025, worldwide revenues obtained from biofertilizers could reach 4.9 billion USD, with an annual growth rate of 11.46% [1]. Several types of microbial inoculants containing nitrogen-fixing microorganisms are now marketed and include symbiotic legume root and stem nodule bacteria (rhizobia) as well as free-living diazotrophic plant growth-promoting rhizobacteria (PGPR), such as *Azospirillum*. In this context, members of the genus *Azospirillum* are among the most studied and used soil microbial inoculants for non-legume crops (e.g., cereals). These inoculants are extensively used because of their growth-promoting functions, mainly due to their ability to fix atmospheric nitrogen (N) and to produce phytohormones (e.g., auxins, gibberellins and cytokinins).

An efficient colonization of crop and pasture plants by inoculated *Azospirillum* spp. is essential to increase yields in agroecosystems. It has been reported that intrinsic factors, such as exo- and lipo-polysaccharide production and motility can be determinants of competence and colonization [2,3]. Other extrinsic factors can also be relevant determinants of efficient colonization by *Azospirillum* spp. In this sense, agricultural practices, such as the N source used can also exert an effect on the colonization of inoculated bacterial strains [4]. In addition, plant genotypes and soil management history affect the structure and functions of the rhizosphere microbial community involved in N cycling [5].

While many of the currently used technologies for enumerating *Azospirillum* spp., and other microbial inoculants, work well in pure culture under laboratory conditions (e.g., *lac*Z, *gus*A, *lux*AB, fluorescent in situ hybridization [FISH] and quantitative PCR [qPCR]), most fail to work adequately under field conditions with complex matrixes due to issues of sensitivity and/or specificity in the soil–plant continuum [6]. It needs to be considered that the limitations of FISH and qPCR are related to the design of strain-specific probes where false positives can occur by the presence of genetically related strains in the soil bacterial communities. Moreover, most currently used technologies to enumerate *Azospirillum* spp. and other PGPB strains under field conditions involve culture-dependent methods, mainly plate-counting on differential or selective agar media. These techniques are laborious and time-consuming procedures (generally from 4 to 7 days), and do not provide data concerning specific strains or the presence of viable but not culturable microbes. Consequently, specific and sensitive methods are required for tracking *Azospirillum* spp. and other microbial inoculants in agriculture, and to better understand the survival and ecological strategies of this bacterium.

More recently, comparative genomic studies have revealed that a large number of prokaryotes (bacteria and archaea) contain clustered regularly interspaced short palindromic repeats (CRISPR) in their genomes. The CRISPR loci have a particular structure [7], where extragenomic DNA (from 28 to 60 bp) is incorporated after first being cleaved. As more extragenomic elements are incorporated, the loci increase their size. New fragments are interspaced by repetitive sequences of similar size (from 28 to 60 bp) providing a fingerprinting specific for each strain, including members of the genus *Azospirillum* [6]. Based on this, we hypothesized that the structure of CRISPR loci could serve as a strain-specific genomic marker for *Azospirillum*. Moreover, we also hypothesized that if this marker could be combined with endpoint PCR, an inexpensive and rapid technique, it may have the potential to be used for control quality and tracking of specific commercial *Azospirillum* strains, and other commercial CRISPR-harboring microbial inoculants. In this study, we report the development of a CRISPR_loci_-PCR approach based on the combined use of a designed primer set for CRISPR loci and a standardized endpoint PCR reaction for the strain-specific detection of *Azospirillum* sp. strain B510 (Figure S1). In addition, we show that the CRISPR_loci_-PCR approach can be used as a tracking tool to identify and examine the distribution of *Azospirillum* sp. strain B510 in two plant niches, the rhizosphere and root endosphere of wheat seedlings.

## 2. Materials and Methods

### 2.1. Bacteria Used in this Study

Detection of CRISPR loci in *Azospirillum* sp. strain genomes was performed by querying the CRISPRdb (https://crispr.i2bc.paris-saclay.fr/ (accessed on 1 June 2017 and January 2020), CRISPI (https://genoweb1.genouest.org/Serveur-GPO/outils/repeatsAnalysis/CRISPR/index.php (accessed on 1 June 2017 and January 2020), and NCBI genome (https://www.ncbi.nlm.nih.gov/genome (accessed on 1 June 2017, 1 January 2020 and 1 May 2021) databases. Thirteen of sixteen *Azospirillum* genomes contained either CRISPR or putative CRISPR loci. *Azospirillum* sp. strain B510 contained two long CRISPR loci, with lengths of 2494 bp and 5243 bp and repeat fragment sizes of 37 bp. Thus, strain B510 (AP010946.1) [8] was selected as target bacterium in this study and was purchased from Japan Collection of Microorganisms (JCM-RIKEN; http://www.jcm.riken.jp (accessed on 1 June 2017). The *Azospirillum* sp. strain B4 (WGS: BACU01000001:BACU01000744) [9] and *Azospirillum* sp. strain B506 (WGS: BADK01000001:BADK01001143) [8], both harboring CRISPR loci, were also acquired from JCM-RIKEN and used as control strains for PCR specificity assays. Additional control strains consisted of *Azospirillum* strains isolated from the wheat rhizosphere in Chile [10], and strains from the genera *Bacillus*, *Microbacterium*, *Chitinophaga*, *Arthrobacter*, *Georgenia*, *Psychrobacillus*, *Roseomonas*, *Bosea* and *Leifsonia* previously isolated from the wheat rhizosphere (20 strains), and endosphere (18 strains) [11]. All bacteria used in this study are listed in Table 1.

### 2.2. Primer Design

**Figure 1:**
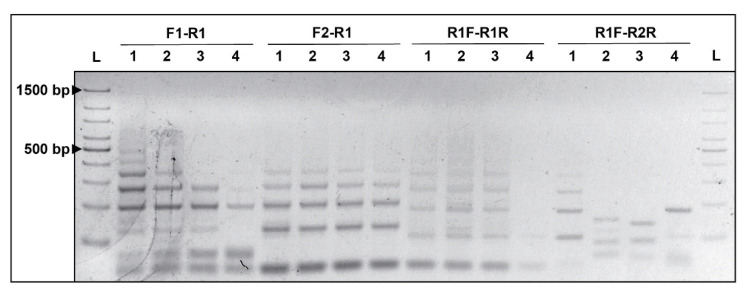
Primer design PCR banding testing from four primer combinations (F1–R1, F2–R1, R1F–R1R; R1F–R2R) on *Azospirillum* sp. strain B510 DNA; L: 100 bp ladder. Lanes 1–4 represent replicates of each primer set PCR reactions.

The CRISPR locus NC_013854_8 in *Azospirillum* sp. strain B510 was selected for primer design. The repeat sequence (5′-GCT TCA ATG AGG CCC AAG CAT TTC TGC CTG GGA AGA C-3′) was used as template for the design of three forward and three reverse primers (Table 2). ‘Fixed’ (FP) and ‘repeat’ (RP) primers were designed, by using Primer3 software [12]. The RP was complementary to a conserved sequence. Four combinations of the six designed primers were established considering the repeat sequence as an annealing zone for the forward primers F1–R1 and F2–R1 (Figure 1) and R1F–R1R and R1F–R2R as the reverse primers (Figure 1). Prior to other standardization and validation experiments, a first PCR assay was conducted for all primer sets using GoTaq^TM^ Flexi DNA polymerase (Promega Inc., Madison, WI, USA), using the manufacturer suggested concentrations and temperatures.

### 2.3. DNA Extraction and PCR Reaction

The purity of strains was checked prior to DNA extraction by streaking on R2A agar plates [13] and incubating at 30 °C for 3 days. One mL of overnight liquid cultures of each strain was used for chromosomal DNA extraction by using the cetyltrimethylammonium bromide (CTAB)-Proteinase K method [14]. The PCR reactions (50 µL) contained 15 ng DNA, 1× PCR Buffer, 1.5 mM MgCl_2_, 1 mM dNTPs, 0.5 mM of repeat targeted primer, 0.5 mM of fixed primer and 0.5 U μL^−1^ Promega GoTaq^TM^ DNA polymerase (Promega Inc.). The PCR conditions were: 95 °C for 7 min, followed by 35 cycles of denaturation at 94 °C for 30 min, annealing at 60 °C for 30 min, extension at 72 °C for 30 min and a final extension at 72 °C for 7 min. PCR products were separated on 1% agarose gels made in TBE buffer and stained with GelRed^®^ 1× Nucleic Acid Gel Stain (Biotium Inc., Fremont, CA, USA).

### 2.4. PCR Reaction Standardization

Improvement of DNA fingerprint patterns was accomplished by varying concentrations of DNA polymerase, primers and dNTPs, changing annealing temperatures and by use of DMSO to enhance denaturation during PCR. All reactions used 15 ng of purified DNA from *Azospirillum* sp. strain B510 as template. Three commercial brands of DNA polymerases were tested: GoTaq^TM^ Flexi (Promega Inc., USA), Invitrogen^TM^ Taq DNA Polymerase (ThermoFisher Scientific Inc., Wartham, MA, USA) and KAPA Taq PCR Kit (KAPA Biosystems, Switzerland). The concentration of primers (IDT Inc., Newark, NJ, USA) and dNTPs (Promega Inc., Madison, WI, USA) tested ranged from 0.1 to 1 µM and from 0.1 to 1 mM, respectively. Repeat-targeted primer (RP) pairs were set at 2× the concentration of the fixed primers (FP). The PCR reactions were run at 95 °C for 10 min, followed by 35 cycles of denaturation at 94 °C for 1 min, annealing from 60 to 65 °C for 1 min, extension at 72 °C for 1 min and final extension of 72 °C for 15 min. Dimethyl sulfoxide (DMSO) was added from 1 to 10% for denaturation enhancement. In addition, the PCR amplicons were visualized using 1.5% of agarose gels prepared with 1% TAE (Tris-acetate-EDTA), TBE (Tris-Borate-EDTA) and/or SB (Sodium borate) buffers, and stained with GelRed^®^ Nucleic Acid Gel Stain 1× (Biotium Inc., San Francisco, CA, USA).

### 2.5. Specificity of CRISPR_loci_-PCR

The specificity of CRISPR_loci_-PCR reactions was evaluated by colony- and endpoint-PCR targeting all strains listed in Table 1. Fifteen ng DNA were used as template for endpoint PCR reactions, whereas for colony PCR reactions, the templates were obtained by scraping colonies from nutrient broth (NB) agar plates cultured at 28 °C for 24 h. Each reaction tube contained 1× PCR buffer, 1.5 mM MgCl_2_, 1 mM dNTPs, 0.1 mM of repeat targeted primer and 0.05 mM of fixed primer, 0.25 U μL^−1^ DNA polymerase and 10% DMSO (only for endpoint PCR reactions). The PCR conditions were: 95 °C for 10 min, followed by 35 cycles of denaturation at 94 °C for 1 min, annealing at 60 °C for 1 min, extension at 72 °C for 1 min and a final extension at 72 °C for 15 min. PCR products were visualized on 1% agarose gels in TBE buffer and stained with GelRed^®^ 1× Nucleic Acid Gel Stain.

### 2.6. Detection Limit of CRISPR_loci_-PCR

The limit of detection of the CRISPR_loci_-PCR assay was ascertained by using *Azospirillum* sp. strain B510 cells that were cultured overnight in nutrient broth at 28 °C, with shaking at 100 rpm. Cells were washed three times and resuspended in sterile saline solution (0.8% NaCl) prior to use. Washed cell suspensions were homogenized and cell numbers determined, in triplicate, by flow cytometry using a Facs Canto II instrument (BD Life Sciences, NJ, USA). In parallel, 1 mL aliquots of cell suspensions were used for triplicate DNA extractions with the DNeasy UltraClean^®^ Microbial Kit (QIAGEN N.V., Düsseldorf, Germany). The concentration of DNA was quantified by using samples that were diluted to 10, 5, 2.5, 1.25 and 0.625 ng DNA in TE buffer. Endpoint PCR was performed using the designed the F1–R1, F2–R1, R1F–R1R and R1F–R2R primer combinations. PCR reactions were performed as described above and DNA patterns were visualized on 1.5% Agarose-TBE gels. The greatest sensitivity (the lowest limit of detection) was observed for the F1–R1 primer set, which was chosen and used in further validation assays.

### 2.7. Validation of CRISPR_loci_-PCR

A wheat seedling assay was used to determine if the CRISPR_loci_-PCR approach could be used as a specific detection tool for *Azospirillum* sp. strain B510 in complex matrixes. Wheat seeds (*Triticum aestivum* (L.) var. Otto) were disinfected for 5 min in NaClO 5%, followed by washing in EtOH 70% for 20 min. Seeds were rinsed three times with sterile distilled water [11] and germinated for 48 h at 20 °C in the dark. In addition, seeds and washing supernatants (50 uL) were plated on LB-agar plates to confirm seed surface sterility. After germination, seeds were sown into substrate (1:1:2 of soil:perlite:peat) and incubated with 16:8 h (22 °C-60% humidity; 16 °C-80% humidity) cycles in a plant growth incubator chamber for 14 days. Sterile substrates were obtained by autoclaving three times at 121 °C × 21 min. The following treatments were investigated: (a) sterile substrate (SS) alone; (b) sterile substrate inoculated with *Azospirillum* sp. strain B510 (SSI); (c) sterile substrate with wheat seedlings (SSW); (d) sterile substrate with wheat seedlings inoculated with *Azospirillum* sp. strain B510 (SSWI); (e) nonsterile (natural) substrate (NS); (f) nonsterile substrate inoculated with *Azospirillum* sp. strain B510 (NI); (g) nonsterile substrate with wheat seedlings (NSW) and (h) nonsterile substrate with wheat seedlings inoculated with *Azospirillum* sp. strain B510 (NSWI).

After incubation, adhered rhizosphere substrates were collected by gently shaking root systems and DNA was extracted from 0.25 g of sample using QIAGEN DNeasy PowerSoil Kit (QIAGEN N.V., Düsseldorf, Germany), following manufacturer instructions. For treatments without plants, 0.25 g of substrate was used for DNA extraction. Root endosphere DNA was also extracted from 0.15 g of root tissue, as previously described [11], using Plant and Seed DNA Miniprep Kits (Zymo Research Inc., Irvine, CA, USA).

PCR was performed on all DNA samples using primer set F1–R1 (Table 2). DNA (15 ng) from *Azospirillum* sp. strain B510 was used as a positive control. Optimized PCR reactions were performed as described above and DNA fragments were visualized on 1.5% Agarose-TBE gels as described above. PCR products were also verified in a 5200 Fragment Analyzer (Agilent Technologies., CA, USA) using the DNF915 dsDNA Reagent kit following manufacturer instructions (35 to 5000 bp) (Agilent Technologies., Santa Clara, CA, USA).

## 3. Results

### 3.1. Primer Design

The primer used for targeting the CRISPR locus NC_013854_8 of *Azospirillum* sp. strain B510 revealed amplification for all designed primer sets using the DNA polymerase manufacturer’s standard protocol (Figure 1). Despite the size of the target locus (2494 bp), no defined bands were detected over 500 bp. Primer sets F1–R1, F2–R1 and R1F–R1R revealed similar banding patterns in terms of band number (four noticeable bands each), and molecular weight. However, the F1–R1 and R1F–R1R combinations presented banding variability between repetitions, with similar size among all reactions. In contrast, the R1F–R2R combination showed differential amplification among repetitions. In addition, the primer combinations F1–R1, F2–R1 and R1F–R2R presented characteristic primer dimer bands, requiring greater standardizations for all tested primer sets. 

### 3.2. CRISPR_loci_-PCR Standardization

**Figure 2:**
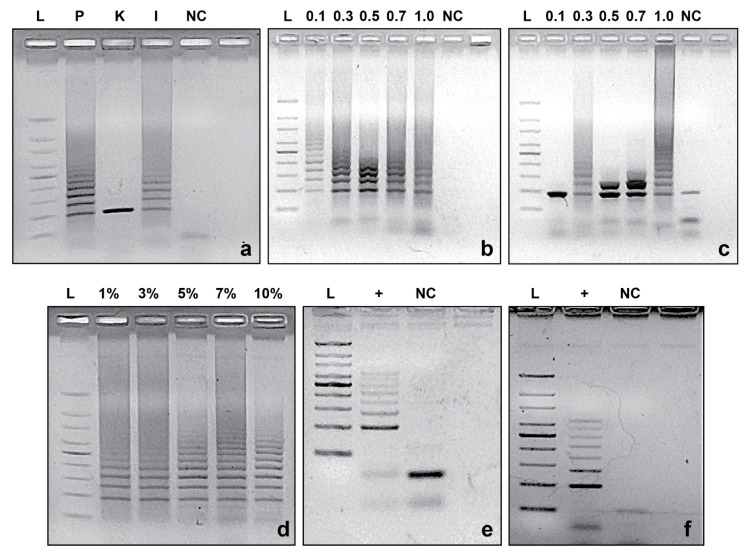
Standardization of CRISPR_loci_-PCR approach using designed F1–R1 primer set. (**a**) Polymerases (P: GoTaq^TM^ Flexi from Promega Inc.; K: KAPA Taq PCR Kit from KAPA Biosystems; I: Invitrogen^TM^ Taq DNA Polymerase from ThermoFisher Scientific Inc.). (**b**) Primer concentrations (µM). (**c**) dNTPs concentrations (mM); (**d**) Dimethyl sulfoxide (DMSO) percentage. Electrophoresis on (**e**) Tris-Borate-EDTA (TBE) and (**f**) Sodium borate (SB) agarose gels. L: 100 bp DNA ladder; NC: negative control; +: positive control (pure DNA from *Azospirillum* sp. strain B510).

CRISPR_loci_-PCR standardization analyzes indicate that Promega GoTaq^TM^ Flexi was the most effective in revealing a sufficient number and types of CRISPR loci in DNA from *Azospirillum* sp. strain B510 (Figure 2a). While a low concentration of primers (0.1 µM RP and 0.05 µM of FP) was found to be optimal to obtain proper and repeatable fingerprints of the targeted CRISPR loci (Figure 2b), a high concentration of dNTPs (as high as 1 mM) was required to reveal sufficient CRISPR loci bands in gels (Figure 2c). 

Among the other parameters examined in the PCR reaction, an optimal annealing temperature of 60 °C provided the best amplification using all primers (Table 2). Interestingly, increasing amounts of DMSO also improved the efficiency of PCR reactions (Figure 2d). The use of 10% DMSO improved band definition and diminished the nonspecific amplification previously observed (Figure 2a–c).

Both the TBE and SB buffers were found to be suitable to reveal the desired CRISPR loci fingerprints (Figure 2e,f, respectively). Although TBE buffer has proven to produce better defined gel bands, SB buffer allowed electrophoresis to be run at twice the voltage in half the time (15 min), with a concomitant reduction in buffer heating.

### 3.3. Specificity of CRISPR_loci_-PCR

**Figure 3:**
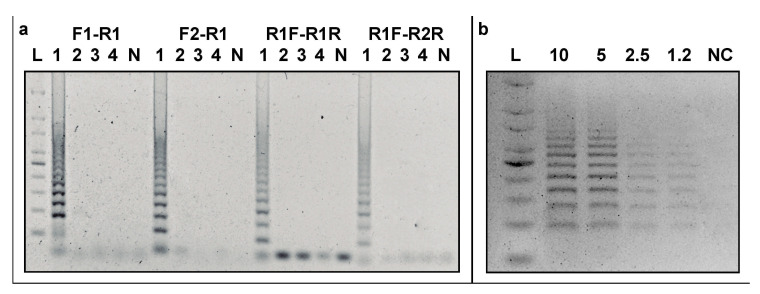
(**a**) Specificity of primer set combinations (F1–R1, F2–R1, R1F–R1R and R1F–R2R) designed in this study: 100 bp DNA ladder; 1: *Azospirillum sp*. strain B510; 2: *Azospirillum* sp. B506; 3: *Azospirillum* sp. B4; 4: *Bacillus* sp. 372EC; N: negative control. (**b**) Detection limit of CRISPR_loci_-PCR approach using F1–R1 primer set with different DNA concentrations of *Azospirillum* sp. strain B510 (10, 5, 2.5 and 1.2 ng DNA). L: ladder; NC: negative control.

Both colony and endpoint PCR assays and template DNA from all the strains listed in Table 1 were used to examine the specificity of the developed CRISPR_loci_-PCR assay. Results of this analysis revealed amplification only for DNA from *Azospirillum* sp. strain B510. No bands were amplified using DNAs from *Azospirillum* sp. strain strains B4 and B506, isolated from the same source as *Azospirillum* sp. strain B510 (Figure 3a), or from seven native *Azospirillum* strains isolated from wheat plants in Chile. Similarly, no CRISPR_loci_-PCR bands were seen using DNAs from any of the other 38 strains tested in this study, representing different genera. 

Agarose gels run using colony PCR only showed bands when strain B510 was used as template, although resolution was remarkably lower when compared to that obtained when using purified DNA as template. The remaining 47 strains assayed did not show amplified CRISPR_loci_-PCR bands under any of the assayed PCR conditions tested. It should be noted, however, that gel analyses revealed a large diffuse band under 100 bp from the samples examined, as is typically characteristic of unutilized primer residue.

### 3.4. Detection Limit

The detection limit for the developed CRISPR_loci_-PCR assay was examined after 24 h of cell incubation. At this time, point cell numbers in culture were 7.49 × 10^6^ cells ml^−1^, as determined by flow cytometry, and this was equivalent to ~15.1 ng µL^−1^ DNA. All four DNA amounts tested (10, 5, 2.5 and 1.25 ng) revealed amplified bands with all the designed primer sets (Figure 3b), although tubes with 2.5 and 1.25 ng of DNA showed non-discernible amplification bands. The lowest amount template DNA tested (0.625 µg) did not result in the production of any visible amplification products. Based on these results, we set the detection limit for the CRISPR_loci_-PCR assay at 5 ng (equivalent to ~2.48 × 10^6^ cells), accomplished using the primers and PCR conditions discussed above.

### 3.5. Method Validation Using Plant Assays

**Figure 4:**
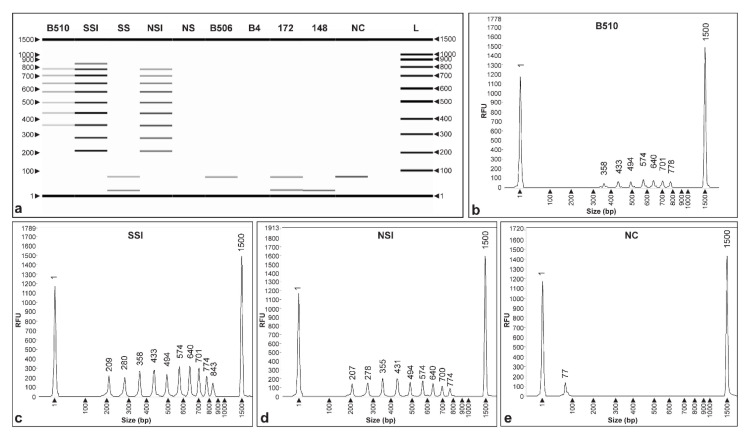
Fragment analysis of CRISPR_loci_-PCR validation assay with substrates. (**a**) Capillary electrophoresis gel of *Azospirillum* sp. strain B510 (positive DNA control), sterile substrate inoculated (SSI), sterile substrate (SS), nonsterile substrate inoculated (NSI), nonsterile substrate (NS); *Azospirillum* spp. (B506, B4, 172, 148), and negative controls (NC). L: 100 bp DNA ladder. Electropherograms of *Azospirillum* sp. strain B510 (**b**), SSI (**c**), NSI (**d**) and NC (**e**).

A two-week wheat plant colonization assay was also conducted to validate the CRISPR_loci_-PCR assay. The samples were collected from plants grown in nonsterile and sterile substrates, and rhizosphere and root endosphere compartments. At sampling time, plants did not show any physiological of phenological differences. Results in Figure 4a show that the CRISPR_loci_-PCR DNA fingerprint banding patterns obtained using plant-derived materials were as expected and similar to those obtained previously. Moreover, *Azospirillum* sp. strain B510-specific DNA fingerprints were seen when using materials obtained from plants grown in sterile and nonsterile substrates (SSI and NSI), and these were comparable to fingerprints obtained when using pure DNA (Figure 4a). In contrast, treatment controls without inoculation (SS and US) did not reveal any amplification bands.

CRISPR_loci_-PCR fragment analysis performed using purified DNA from *Azospirillum* sp. strain B510 and the SSI treatment revealed the molecular weight of the fragments to be 358, 433, 494, 574, 640, 701 and 778 (Figure 4b), and 209, 280, 358, 433, 494, 574, 640, 701, 774 and 843 bp (Figure 4c), respectively. Similarly, fragment analyses realized using DNA from the NSI treatment produced similar sized fragments to pure DNA, with 1–2 bp slight molecular weight variation with fragments sizes of 207, 278, 355, 431, 494, 574, 640, 700 and 774 bp (Figure 4d). None of the negative controls (DNA from strains B506, B4, 172, 148 and NC) revealed peaks using fragment analysis columns or bands on agarose gels (Figure 4a,e).

**Figure 5:**
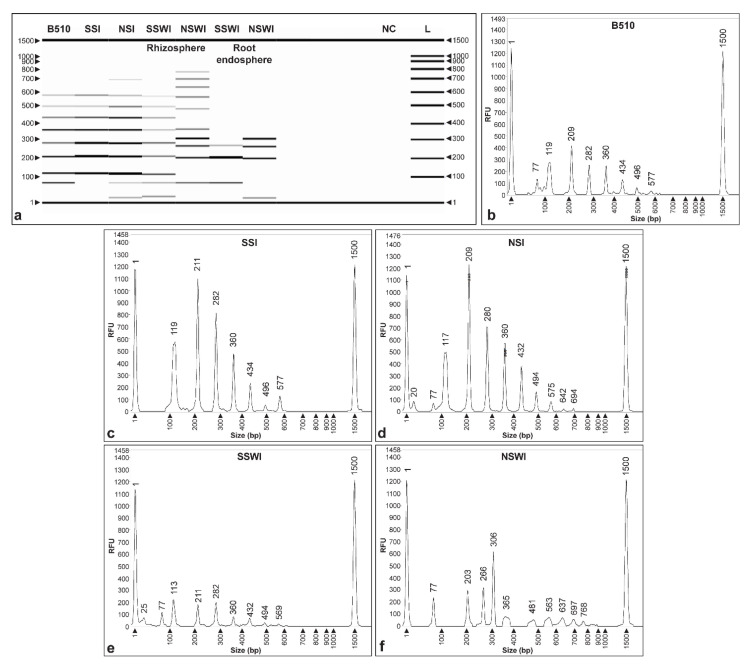
Fragment analysis of CRISPR_loci_-PCR validation assay with plants. (**a**) Capillary electrophoresis gel *Azospirillum* sp. strain B510 (positive DNA control), sterile substrate inoculated (SSI), nonsterile substrate inoculated (NSI), sterile substrate with wheat seedling inoculated with *Azospirillum* sp. strain B510 (SSWI) and nonsterile substrate with wheat seedling inoculated with *Azospirillum* sp. strain B510 (NSWI); L: 100 bp DNA ladder. Electropherograms of *Azospirillum* sp. strain B510 (**b**), SSI (**c**), NSI (**d**), SSWI (**e**) and NSWI (**f**).

Interestingly, PCR assays not only showed similar banding patterns when compared with *Azospirillum* sp. strain B510 using DNA extracts from inoculated sterile (SSI) and nonsterile substrates (NSI), but also using rhizosphere and endosphere samples (Figure 5), although the banding observed started at smaller molecular size (~ 100 bp) if compared (Figure 4a). In particular, the rhizosphere of seedlings grown in sterile substrate (SSWI) showed similar fingerprint (113, 211, 282, 360, 432 and 569 bp; Figure 5e) compared with *Azospirillum* sp. strain B510 (Figure 5b). However, rhizosphere samples from seedlings grown in nonsterile substrate (NSWI) presented absence, or some dissimilarities, in bands (203, 266, 306, 365, 481, 563, 637, 697 and 768 bp) (Figure 5f). In endosphere samples, electrophoresis only revealed three and four fragments consistent with the DNA fingerprints of *Azospirillum* sp. strain B510 (Figure 5a).

## 4. Discussion

Tracking the fate of biofertilizer-based microbial inoculants released into the environment is a fundamental aspect of their technological success. Assessing the colonization and in situ activity of an inoculated microbe has been the bottleneck of biofertilizer studies since the first inoculation trials [15]. The former needs to be addressed in order to reduce the significant yield differential between chemical fertilization and biofertilization.

Hereby, we used the CRISPR locus repeat sequence NC_013854 (5′-GCT TCA ATG AGG CCC AAG CAT TTC TGC CTG GGA AGA C-3′) to developed four primer sets that specifically targeted *Azospirillum* sp. strain B510. The specificity of such primers was tested *in silico* under NCBI BLASTn (http://blast.ncbi.nlm.nih.gov (accessed on 1 June 2017, January 2020, and January 2021), where the selected CRISPR-repeat sequence did not reveal similarity with other organisms or metagenomic datasets sequenced to date. The four primer sets demonstrated to be specific; however, the primer set F1–R1 (5′-CGA ACG CTT TCT CAA ACC AC-3′ and 5′-AGA AAT GCT TGG GCC TCA TT-3′, respectively) demonstrated the ability to produce the most reliable amplification at low concentrations of purified DNA (~1.2 ng μL).

After standardization, fingerprint analyses were performed using genomic DNA from other two culture collection *Azospirillum* sp. CRISPR-harboring strains, as well as nine other native *Azospirillum* spp., and 38 native strains of different taxa previously isolated from wheat rhizosphere and root endosphere from the Region de La Araucanía [10,11].

To achieve strain-specificity and enhance PCR performance in this study, several prior steps were considered. The identification of CRISPR loci by our method relies exclusively in the availability of a completely and properly assembled genome, which needs to be further analyzed. Currently, the CRISPR databases are in constant curation [16], nonetheless genome databases have a much faster growth rate and therefore external software such as PILER-CR [17] can be used to detect CRISPRs without the use of specific databases. During the experimental designs, however, it is important to note that the target CRISPR repeats usually present palindromic sequences [18], and thus several primer combinations are required for the design to be successful.

Comparable PCR-based approaches to those proposed in this study have also been reported. Three primer sets targeting sequence characterized amplified region (SCAR) markers were designed and implemented for endpoint and qPCR assays for the quality control of a biofertilizer consortium composed of *Azotobacter chroococcum*, *Bacillus megaterium* and *Azospirillum brasilense* strains [19]. While authors described this method as reliable for field applications, once the designed primer sequences are subjected to BLASTn *in silico* analyses, cross reactivity and false positive biases with other plant–soil associated bacteria are found. Similar results were observed on a qPCR SCAR markers assay for *Pseudomonas brassicacearum* MA250 [20] and *Azospirillum. lipoferum* CRT1 [21]. Despite the primer specificity for such SCAR genes, strain-specificity could not be assured. By contrast, our CRISPR loci-based method development first considered the strain specificity of the DNA primer sets prior to their evaluation in plant assays.

In this study, the selected *Azospirillum* sp. strain B510 was detected by conventional PCR in sterile and nonsterile substrate, as well as in the rhizosphere of wheat seedlings sown in sterile substrate, under growth chamber conditions. It needs to be addressed that, despite differences in band size detected between the two PCR validation assays, all assays using this methodology should be accomplished in concert with purified DNA from the inoculum purified DNA as a control. Complementarily, all novel PCR assays based in this approach must be appropriately standardized by using several combinations and brands of reagents for every targeted bacterial strain because strain-specific PCR reaction conditions might significantly vary. It has been widely reported the biases in PCR-based methods produced using complex environmental samples, such as soil and rhizosphere soils, may be due to a low efficiently of DNA extraction, PCR inhibitor (e.g., humic and fulvic acids), etc. [22] In addition, plant rhizosphere is recognized as a complex matrix that harbor DNA from thousands of microbial taxa and as one of the main hotspots for activity of microorganisms in terrestrial ecosystems [23]. This high complexity could have affected the efficiency and specificity of designed primer sets using CRISPR_loci_-PCR. This problem could have also been exacerbated in the root endosphere samples, considering that not only microbial DNA is extracted but also a higher portion of DNA belongs to plant tissues, producing unspecific PCR reactions. It is necessary to mention that the DNA extraction kit used for endosphere samples here was designed for plant DNA; however, specific methods for the extraction of endosphere bacteria are scarce and difficult to implement due to the plant tissue’s internal biochemistry, as well as differences between plant species [24]. In addition, the fingerprinting differences we have seen may due to low levels of colonization, or possibly the absence of inoculated strain in this plant niche. In this sense, studies have revealed that plant endospheres recruit specific microbial communities which can significantly differ from those found in rhizospheres of the same plant [25]. There are multiple factors that could be involved, such as the number of cells that reside in the host plant root tissues, interferent plant DNA present in the sample or incompatibility between the inoculum and the host. 

The strain specificity observed in the PCR reactions confirms that the designed primer set, as well as the target CRISPR loci could be used for tracking of microbial inoculants, but further and deeper studies are still required to use the CRISPR_loci_-PCR approach at field level. Moreover, it must be taken into consideration that environmental and ecological studies accomplished using CRISPR loci are scarce in samples obtained from agricultural soils. Among those, the nearest approximation to our design is a CRISPR-based typing study of 85 *Erwinia amilovora* strains [26].

Finally, in this study we used CRISPR loci to develop an inexpensive and easy-to-apply method for a specific *Azospirillum* strain, one of the most used bacterial genera in agriculture. The CRISPR_loci_-PCR approach proposed here might be used as template for further initiatives directed to evaluate the quality of commercial biofertilizers, their colonization and persistence, once commercial *Azospirillum* strains are inoculated by farmers and technical laboratories. In its current state, this PCR approach may also be implemented via higher-throughput PCR variants (such as quantitative PCR and droplet digital PCR) and cultivation of bacteria (such as microcolonies counting), and therefore replace the use of conventional plate-counting methods, which are a time-consuming and strain-unspecific approaches.

## Figures and Tables

**Table 1:** Bacterial strains used in this study.

Strain	Plant Niche Source	Country	16S Accession No. ^a^	Genome Accession No. ^b^	Reference
**Target bacterium**					
*Azospirillum* sp. B510	Rice stems endosphere	Japan	AB049111	AP010946.1	Elbetagy et al. 2001
**Azospirillum control strains**					
*Azospirillum* sp. B506	Rice stems endosphere	Japan	AB049110	BADK01000001:BADK01001143	Elbetagy et al. 2001
*Azospirillum* sp. B4	Rice stems endosphere	Japan	AB027690	BACU01000001:BACU01000744	Elbetagy et al. 2000
*Azospirillum* sp. I53	Wheat rhizosphere	Chile	MK216930	n.s.	Acuña et al. 2020
*Azospirillum* sp. I84	Wheat rhizosphere	Chile	MK216960	n.s.	
*Azospirillum* sp. I118	Wheat rhizosphere	Chile	MK216988	n.s.	
*Azospirillum* sp. I146	Wheat rhizosphere	Chile	MK217013	n.s.	
*Azospirillum* sp. I148	Wheat rhizosphere	Chile	MK217015	n.s.	
*Azospirillum* sp. I171	Wheat rhizosphere	Chile	MK217036	n.s.	
*Azospirillum* sp. I51	Wheat rhizosphere	Chile	MK216928	n.s.	
**Control strains**					
*Microbacterium* sp. 62CR	Wheat rhizosphere	Chile	MG835569	n.s.	Rilling et al. 2018
*Bacillus* sp. 72CR	Wheat rhizosphere	Chile	MG835570	n.s.	
*Bacillus* sp. 102CR	Wheat rhizosphere	Chile	MG835571	n.s.	
*Bacillus* sp. 112BR	Wheat rhizosphere	Chile	MG835572	n.s.	
*Bacillus* sp. 154AR	Wheat rhizosphere	Chile	MG835573	n.s.	
*Bacillus* sp. 173CR	Wheat rhizosphere	Chile	MG835574	n.s.	
*Microbacterium* sp. 184AR	Wheat rhizosphere	Chile	MG835575	n.s.	
*Bacillus* sp. 184AR-1	Wheat rhizosphere	Chile	MG835576	n.s.	
*Bacillus* sp. 214AR	Wheat rhizosphere	Chile	MG835577	n.s.	
*Bacillus* sp. 214AR-1	Wheat rhizosphere	Chile	MG835578	n.s.	
*Bacillus* sp. 222BR	Wheat rhizosphere	Chile	MG835579	n.s.	
*Chitinophaga* sp. 623EA	Wheat rhizosphere	Chile	MG835588	n.s.	
*Arthrobacter* sp. 243AR	Wheat rhizosphere	Chile	MG835580	n.s.	
*Bacillus* sp. 322CR	Wheat rhizosphere	Chile	MG835583	n.s.	
*Bacillus* sp. 342CR	Wheat rhizosphere	Chile	MG835584	n.s.	
*Microbacterium* sp. 354AR	Wheat rhizosphere	Chile	MG835585	n.s.	
*Bacillus* sp. 354AR-1	Wheat rhizosphere	Chile	MG835586	n.s.	
*Bacillus* sp. 372EC	Wheat rhizosphere	Chile	MG835587	n.s.	
*Bacillus* sp. 503CR	Wheat rhizosphere	Chile	MG835581	n.s.	
*Bacillus* sp. 503CR-1	Wheat rhizosphere	Chile	MG835582	n.s.	
*Mycobacterium* sp. 222EC	Wheat root endosphere	Chile	MG835593	n.s.	
*Microbacterium* sp. 382EC	Wheat root endosphere	Chile	MG835590	n.s.	
*Georgenia* sp. 424EC	Wheat root endosphere	Chile	MG835591	n.s.	
*Psychrobacillus* sp. 444EC	Wheat root endosphere	Chile	MG835592	n.s.	
*Mycobacterium* sp. 491EC	Wheat root endosphere	Chile	MG835594	n.s.	
*Roseomonas* sp. 513EC	Wheat root endosphere	Chile	MG835595	n.s.	
*Roseomonas* sp. 523EC	Wheat root endosphere	Chile	MG835596	n.s.	
*Roseomonas* sp. 543EC	Wheat root endosphere	Chile	MG835598	n.s.	
*Bacillus* sp. 564EB	Wheat root endosphere	Chile	MG835599	n.s.	
*Bacillus* sp. 584EA-1	Wheat root endosphere	Chile	MG835600	n.s.	
*Bacillus* sp. 592BR	Wheat root endosphere	Chile	MG835601	n.s.	
*Chitinophaga* sp. 643EA	Wheat root endosphere	Chile	MG835602	n.s.	
*Bosea* sp. 693EB	Wheat root endosphere	Chile	MG835603	n.s.	
*Bosea* sp. 703EB	Wheat root endosphere	Chile	MG835604	n.s.	
*Leifsonia* sp. 274EB	Wheat root endosphere	Chile	MG835589	n.s.	
*Mycobacterium* sp. 222EC	Wheat root endosphere	Chile	MG835593	n.s.	
*Bacillus* sp. 714EA	Wheat root endosphere	Chile	MG835605	n.s.	
*Georgenia* sp. 734EA	Wheat root endosphere	Chile	MG835606	n.s.	

^a^ Accession number of 16S RNA gene sequences in NCBI GenBank nucleotide database (http://ncbi.nlm.nih.gov/nucleotide (accessed on June 2017). ^b^ Accession number of WGS present in NCBI genome database (http://ncbi.nlm.nih.gov/genome (accessed on June 2021); n.s.: without genome sequences available.

**Table 2:** Primer oligos designed for this study.

Primer Name	Sequence (5′-3′)	Melting Temperature (°C)	Fixed or Repeat
**Forward**			
F1	CGAACGCTTTCTCAAACCAC	60.81	Fixed
F2	CCTTTTCTCTCACACACGCTAC	59.05	Fixed
R1F	ATGAGGCCCAAGCATTTCTG	62.43	Repeat
**Reverse**			
R1	AGAAATGCTTGGGCCTCATT	60.96	Repeat
R1R	CCACTTCGACAGCTTTCTCC	59.99	Fixed
R2R	AGGCCCAAGCATTTCTGC	61.30	Fixed

## Data Availability

16S and WGS sequences reported in Table 1 are available in NCBI GenBank and Genome databases (http://ncbi.nlm.nih.gov (accessed on 1 June 2017 and January 2020, May 2021). The CRISPR-loci selected for primer design (NC_013854_8) is available in https://crispr.i2bc.paris-saclay.fr (accessed on 1 June 2017 and January 2020). Commercial *Azospirillum* sp. strains B510, B4 and B506 are available for acquisition in JCM-RIKEN culture collection.

## Supplementary Materials

The following are available online at https://www.mdpi.com/article/10.3390/microorganisms9071351/s1, Figure S1: Proposed strategy for the tracking of a specific *Azospirillum* strain based on combined use of PCR and CRISPR loci structure. (a) Primers designed for CRISPR_loci_-PCR approach using fixed primer (FP) as forward primer and repeat primer (RP; complementary to conserved sequence repeats) as reverse primer. (b) Primers designed for CRISPR_loci_-PCR approach using repeat primer (RP; complementary to conserved sequence repeats) as forward primer and fixed primer (FP) as reverse primer. Target bacterium is named as “Inoculant”.
