# Supplementary material for: CRISPR loci-PCR as Tool for Tracking Azospirillum sp. Strain B510
Source: Microorganisms. 2021 Jun 22;9(7):1351. doi: 10.3390/microorganisms9071351 (PMC8307223; doi:10.3390/microorganisms9071351)
Supplement: Supplementary file 1 [file microorganisms-09-01351-s001.zip › microorganisms-1239029-supplementary.pdf]

microorganisms

MDPI

Supplementary Material

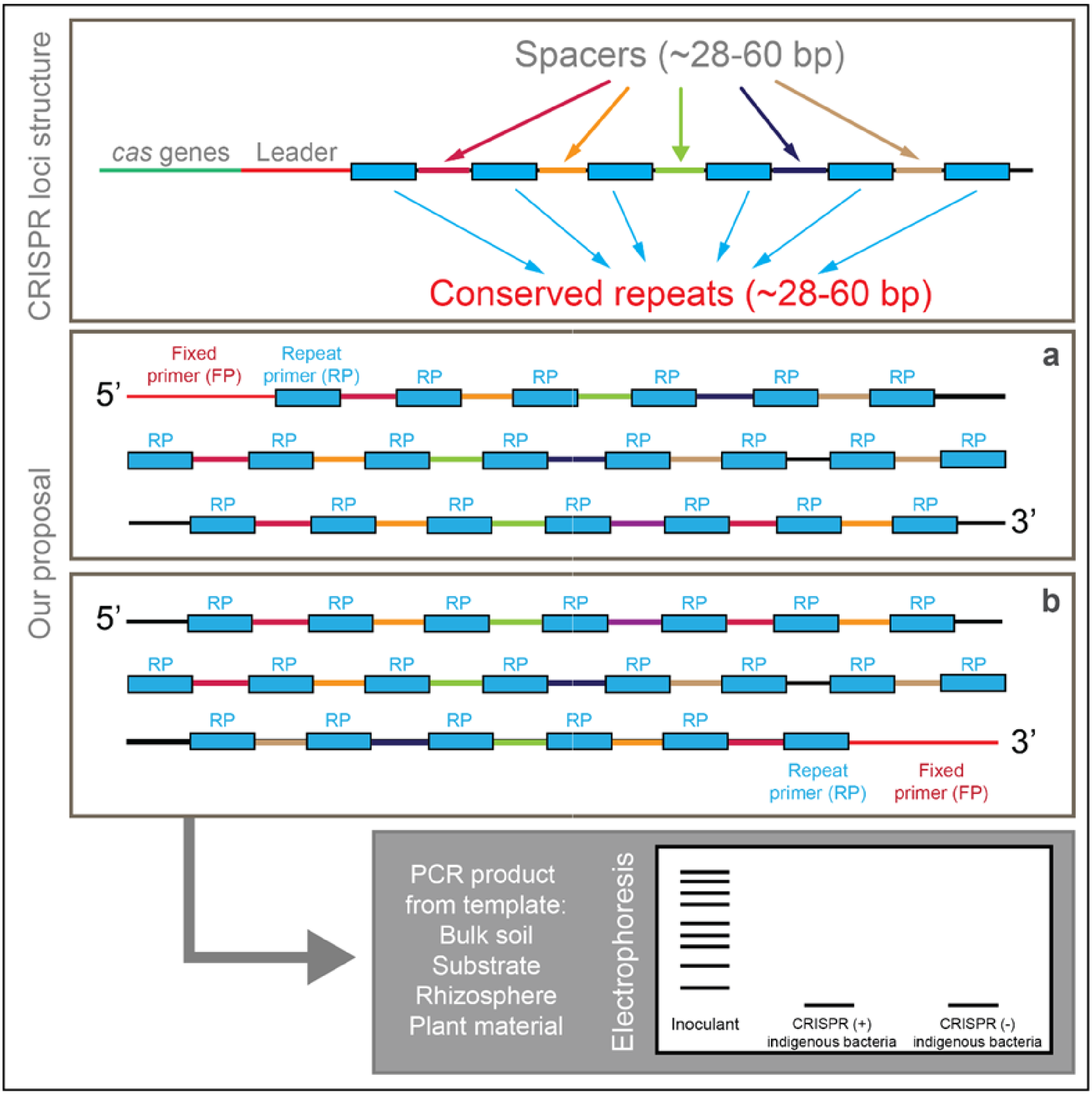

Figure S1. Proposed strategy for the tracking of a specific *Azospirillum* strain based on combined use of PCR and CRISPR loci structure. (a) Primers designed for CRISPR$_{loci}$–PCR approach using fixed primer (FP) as forward primer and repeat primer (RP; complementary to conserved sequence repeats) as reverse primer. (b) Primers designed for CRISPR$_{loci}$–PCR approach using repeat primer (RP; complementary to conserved sequence repeats) as forward primer and fixed primer (FP) as reverse primer. Target bacterium is named as "Inoculant".
